# Navigation on temporal networks

**DOI:** 10.1007/s41109-025-00697-9

**Published:** 2025-03-20

**Authors:** Omar F. Robledo, Petter Holme, Huijuan Wang

**Affiliations:** 1 Faculty of Electrical Engineering, Mathematics, and Computer Science, Delft University of Technology, Delft, The Netherlands; 2 Department of Computer Science, Aalto University, Espoo, Finland; 3 Center for Computational Social Science, Kobe University, Kobe, Japan

## Abstract

Temporal networks, whose network topology changes over time, are used to represent, e.g., opportunistic mobile networks, vehicle networks, and social contact networks, where two mobile devices (autos or individuals) are connected only when they are close to (interact with) each other. Such networks facilitate the transfer of information. In this paper, we address the problem of navigation on temporal networks: how to route a traffic demand from a source *s* to a destination *d* at time $t_s$, based on the network observed before $t_s$? Whenever the node hosting the information has a contact or interacts with another node, the routing method has to decide whether the information should be forwarded to the contacted node or not. Once the information is forwarded, the contacted node becomes the only node hosting the information. Firstly, we introduce a framework of designing navigation algorithms, in which a distance metric is defined and computed between any node to the target *d* based on the network observed before $t_s$. Whenever a hosting node has a contact, it forwards the information to the contacted node if the contacted node is closer to the target than the hosting node according to the distance metric. Secondly, we propose systematically distance metrics of a node pair in the temporal network observed, that capture different network properties of a node pair. Thirdly, these metrics or routing strategies are evaluated in empirical contact networks, from the perspective of the time duration of the routing and the probability that the destination can be reached. Their performance is further explained via the correlation between distance metrics and the stability of each metric in ranking nodes’ distance to a target node. This work may serve as inspiration for evaluating and redesigning these strategies in other types of networks beyond physical contact networks.

## Introduction

Networks (Newman 2010) are commonly used to represent complex systems, with the elements being the nodes of the network, and interactions or relations between them, the links. The transfer of information is a vital function in many real and artificial complex systems, such as the Internet, social, transportation, or telecommunication networks, and knowing the whole structure might not be possible. Navigation is the problem of transferring, or routing, this information with partial structure of the underlying network.

Many real-world networks evolve over time and are thus called temporal networks. Examples include opportunistic mobile networks, vehicle networks, and social contact networks, where two mobile devices (autos or individuals) are connected only when they are close to (interact with) each other. In these networks, information is transported along time-respecting paths (Holme and Saramäki 2012), meaning that each link in the chain from one node to another has to occur or be active at a later time than the previous ones. For example, two mobile devices in an opportunistic mobile network could communicate with each other via Bluetooth or WiFi when they are within each other’s transmission range (Fall 2003). Information such as multimedia newspapers and movie trailers that do not have strict real-time constraints can be transported in such opportunistic mobile networks, offloading cellular traffic. Moreover, temporal networks show properties related to their evolving nature, such as the heavy-tail distribution of inter-event times between consecutive interactions (*burstiness*) or the heterogeneous distribution of the number of interactions between a node pair. We argue that said temporal properties not only affect how information is transported through the network, but that they could be used to improve the routing of information in a temporal network with only partial knowledge of its structure.

The problem of navigation has been widely studied in static networks (see Kleinberg 2000; Kumar et al. 2006; Şimşek and Jensen 2008; Boguñá et al. 2009; Papadopoulos et al. 2010; Kleineberg and Helbing 2017; Yan et al. 2020). Several studies have shown that efficient network navigation can be achieved without knowledge of the global structure of the network; e.g., by using greedy algorithms, such as the ones proposed by Şimşek and Jensen (2008) or Papadopoulos et al. (2010). Ortiz et al. (2017) showed how temporal networks can be navigated more efficiently than static networks. Lee and Holme (2019) proposed intuitive greedy algorithms for navigation in temporal networks, and studied their effectiveness, in terms of hop-count and time, in multiple empirical temporal networks.

In this paper, we will focus on the problem of navigation on temporal networks, as previously studied by Lee and Holme (2019), and interpret it as a *routing* problem; i.e., we study how we can transfer information from a source node *s* to a target node *t* starting at a particular time $t_s$. Whenever there is a contact involving the node in which the information is held, we have to decide whether the information should be forwarded via the contact to another node or remain at the current node. Once a node has forwarded the information package, it deletes the record to conserve storage space. During this navigation process of the traffic demand $(s,d,t_s)$, only one node holds the information at any time. We aim to design effective navigation methods that allow each hosting node to decide whether it should forward the information or not to the node it contacts now, using the temporal network observed before the starting time $t_s$ of the traffic demand $(s,d,t_s)$. We define a framework for designing navigation algorithms in which a distance metric is evaluated between any node and the target node *d*. Whenever there is a contact between a hosting node and a contacted node, the information is transported to the contacted node if the contacted node is closer to the target node than the hosting node according to the distance metric. We propose several distance metrics/properties of a node pair in the temporal network observed. They capture different network properties of a node pair in the time-aggregated network of the observed temporal network or the observed temporal network. We evaluate these metrics or, equivalently, navigation algorithms based on the probability that a target node can be reached and the average time to reach a target node. Traffic demands between all possible node pairs starting at various times in various physical contact networks have been considered in the evaluation. The proposed navigation methods are also compared to the optimal routing in the ideal case in which the future temporal network is known and used to route the information optimally. The optimal routing routes any traffic demand via the fastest time-respecting path. Its performance is, thus, an upper bound for any navigation algorithm. Finally, we study the differences between the performance of these metrics in different empirical networks and the stability of the metrics over time.

The remainder of the paper is structured as follows. In Sect. 2, we describe our approach, detailing our evaluation process, the properties of the data sets used, and the baselines with which we compare our framework. In Sect. 3, we analyse the performance of the navigation strategies proposed and that of the baselines, and compare them. We also study the rank stability of each link at different time steps by using a specific metric. Finally, we present our conclusions in Sect. 4.

## Methods

We aim to develop a generic framework to design navigation algorithms on a temporal network by using past information of the network. First, we introduce the representation of temporal networks. Second, the navigation problem and our evaluation method for the navigation strategies are defined. Third, we describe the empirical contact networks used for the evaluation. Finally, we describe our framework, under which we introduce baseline navigation strategies, and propose our strategies.

### Temporal network representation

A temporal network measured at discrete times can be represented as a sequence of network snapshots $G=\{G_1, G_2,..., G_T\}$, where *T* is the duration of the observation window [1, *T*] and $G_t=(V; E_t)$ is the snapshot at time step *t* with *V* and $E_t$ being the set of nodes and contacts, respectively. If two nodes, *j* and *k*, have a contact at time step *t*, $(j,k)\in E_t$. Here, we assume all snapshots share the same set of nodes *V*, of size *N*. The temporal network *G* can be represented by a *temporal adjacency matrix* *A*, an $N \times N \times T$ matrix in which $a_{i,j,t} = 1$ if node *i* and *j* are connected or have a contact at time *t*, and $a_{i,j,t} = 0$ otherwise. The corresponding time-aggregated network $G^w$ contains the same set of nodes *V* and the set of links $E=\cup _{t=1}^TE_t$. A pair of nodes is connected with a link in the aggregated network if at least one contact occurs between them in the temporal network. The total number of links is $M=|E|$. The weight of each link in the aggregated network is the total number of contacts occurring along the link within [1, *T*].

### Evaluation of navigation methods

We focus on the following navigation problem on a temporal network. Given a traffic demand $(s,d,t_s)$, the information initially hosted at node *s* at time $t_s$ needs to be navigated to the destination *d*. The objective is to design a navigation strategy that decides, whenever a hosting node has a contact with any other node *k*, whether the hosting node should forward the information to *k* or not (thus waiting for its later contact), based on the temporal network observed before $t_s$, thus observed within $[1,t_s]$. Once the information is forwarded to, e.g., *k*, only *k* hosts the information, and nodes that have previously hosted the message have no record of the message anymore. This navigation problem corresponds to the routing problems in temporal networks, e.g., opportunistic mobile networks, vehicle networks, and social contact networks, when the storage capacity of information at nodes is limited.

Given a network, we consider traffic demands from any node to any other node starting at various starting times chosen uniformly from 1 to *T* to evaluate the performance of any navigation strategy *X*. The performance of navigation strategies is evaluated from two perspectives, efficiency with respect to the time duration of the routing paths and effectiveness regarding the probability that a feasible routing path can be found.

For each starting time $t_s$, we define the *efficiency* of any navigation strategy *X* in transporting traffic demands starting at $t_s$ between all source and destination node pairs on a temporal network $\mathcal {G}$ as

1 $$\begin{aligned} E_{\mathcal {G}}^{X}(t_s) = \dfrac{1}{|\Psi (t_s)|} \sum _{s,d \in \Psi (t_s)} \dfrac{\tau _{s,d}(t_s)}{\tau _{s,d}^X(t_s)}, \end{aligned}$$

where $\tau _{s,d}^{X}(t_s)$ is the temporal distance of the path to route traffic demand $(s,d,t_s)$ by following strategy *X*, i.e., the time when the information reaches the destination *d* by using strategy *X* minus $t_s$; $\tau _{s,d}(t_s)$ is the temporal distance of the optimal path in the ideal case where future contacts are known and traffic is routed along the fastest time-respecting path and the set $\Psi (t_s)$ contains each source and destination node pair, between which since $t_s$ a time-respecting path exists in network *G*, i.e. a feasible path can be found by the optimal routing based on the past and future temporal network. For any node pair $s,d \in \Psi (t_s)$, $\tau _{s,d}(t_s)$ is finite. If the traffic demand $(s,d,t_s)$ cannot be met, i.e., the destination cannot be reached according to *X*, the temporal distance $\tau _{s,d}^{X}(t_s)$ is infinity. The efficiency $E_{\mathcal {G}}^{X}({t_s})\in [0, 1]$, with 1 meaning that the strategy performs optimally, i.e., as well as in the ideal case where future contacts are known. A smaller efficiency implies that the strategy performs far below the optimal routing when the future network is known. This metric is inspired by the *temporal closeness centrality*, defined by Pan and Saramäki (2011).

Beyond efficiency, we also evaluate the performance of navigation methods via the probability that a traffic demand is met, i.e., the target could be reached, starting at a given time. We define the *effectiveness* of navigation strategy *X* as

2 $$\begin{aligned} \varepsilon _{\mathcal {G}}^{X}(t_s) = \dfrac{|\Psi ^{X}(t_s)|}{|\Psi (t_s)|}, \end{aligned}$$

where the set $\Psi ^{X}(t_s)$ contains each source destination node pair (*k*, *m*), such that, starting from node *k* at $t_s$, node *m* can be reached by following strategy *X*. Similar to the *efficiency* defined above, the *effectiveness* $\varepsilon _{\mathcal {G}}^{X}(t_s) \in [0, 1]$, with 1 meaning that for any traffic demand starting at $t_s$, the strategy can find a feasible path, i.e., a time-respecting path, as long as the optimal routing can find one.

### Navigation strategies

Given a traffic demand $(s,d,t_s)$ and the temporal network observed before the starting time $t_s$, a navigation method is used to navigate the traffic demand. Specifically, each hosting node uses the navigation method to decide whether to forward the information or not when it contacts another node.

#### Baseline strategies

First, we review baseline strategies (Lee and Holme 2019) that do not make use of previous information of the network.

*Wait for Target (WFT)* Once the navigation process has started, the information is forwarded only if the hosting node has a direct contact with the target node.

*Greedy Walk (GW)* The information is transferred to a different node every time there is a contact. In case a hosting node has multiple contacts at the same time stamp, the information is transported to one of the contacted nodes, chosen uniformly at random.

#### Proposed strategies

Our methodology differs from the aforementioned baselines in that previous information of the network is considered to inform the navigation. We define the following framework of designing navigation algorithms. Given the traffic demand $(s,d,t_s)$, a distance metric is derived between any node to the target node *d* based on the temporal network observed before $t_s$. Whenever there is a contact between a hosting node and a contacted node, the information is forwarded to the contacted node if the contacted node is closer to the target node than the hosting node according to the distance metric.

We will propose various definitions of distance metric $m^X_{i,d}(t_s)$ from node *i* to target node *d* starting at time $t_s$, using contacts occurred prior to the starting $t_s$ of the navigation. In this notation, *X* denotes the specific metric or strategy used. This metric is computed once before the navigation starts. We propose three metrics, each capturing a different aspect of the network and leading to a navigation strategy. In order to compute them, we consider the temporal network observed before $t_s$, i.e., $G^*{(t_s)}=\{G_1, G_2,\ldots , G_{(t_s)}\}$, where $t_s<T$. The time aggregated network $G^*_w{(t_s)}$ of $G^*{(t_s)}$ is a weighted network and can be represented by its weighted adjacency matrix $W(t_s)$ with the weight $w_{i,j}({t_s})$ being the total number of contacts that occur between *i* and *j* during $[1,t_s]$.

*Effective Resistance (RES)* We propose the effective resistance $m^{RES}_{i, d}(t_s)$ between two nodes in the time aggregated network $G^*_w{(t_s)}$. It is the effective resistance between the two nodes in the electronic circuit that is constructed by considering every link (i,j) in the aggregated network $G^*_w{(t_s)}$ as a resistor, whose impedance is 1 over its weight $w_{i,j}({t_s})$. The longer the paths between two nodes, the higher the effective resistance; similarly, the more paths existing between two nodes, the lower the effective resistance between them.

The *Laplacian* matrix corresponding to the aggregated network $G^*_w{(t_s)}$ is defined as $L(t_s) = D(t_s) - W(t_s)$, with $D(t_s)$ being the diagonal matrix, such that $D_{i,i}(t_s) = \sum _{j=1}^{N} w_{i,j}(t_s)$. Mathematically, the effective resistance between nodes *i* and *d* in $G^*_w{(t_s)}$ can be derived as:

3 $$\begin{aligned} m^{RES}_{i, d}(t_s) = L^{-1}_{i,i}(t_s) - 2 \cdot L^{-1}_{i,d}(t_s) + L^{-1}_{d,d}(t_s), \end{aligned}$$

with $L^{-1}$ being the pseudo-inverse of the Laplacian $L(t_s)$. It is important to note that this distance metric $m^{RES}_{i, d}(t_s)$ does not use any temporal information, as it is based on the aggregated topology, but takes into account multiple possible paths in the aggregated network observed.

*Average Time to Target (T2T)* To design this metric, we utilise the temporal network observed so far $$G^*{(t_s)}$$ till the moment $$t_s$$ when a new traffic demand needs to be routed. If traffic demands from *i* to *d* starting before $$t_s$$ can be transported on average within a short time in $$G^*{(t_s)}$$, the distance metric $$m_{i,d}^{T2T}(t_s)$$ is supposed to be small. Given a traffic demand $$(i,d,t_k)$$, where $$t_k<t_s$$, this strategy considers the fastest time-respecting path from node *i* to node *d* in $$G^*{(t_s)}$$, and its temporal distance $$\tau _{i,d}(t_k)$$. If node *d* cannot be reached in $$[t_k, t_s)$$, then $$\tau _{i,d}(t_k) = \infty$$.

The average time to target $m_{i,d}^{T2T}(t_s)$ is the average temporal distance of the fastest time-respecting paths from node *i* to target node *d* starting at times $t_k\in [1, t_s)$ that finish before $t_s$, i.e.,

4 $$\begin{aligned} m_{i,d}^{T2T}(t_s) = \dfrac{1}{|\mathcal {T}_{i,d}^*(t_s)|} \sum _{t_k \in \mathcal {T}_{i,d}^*(t_s)} \tau _{i,d}(t_k), \end{aligned}$$

with $\mathcal {T}_{i,d}^*(t_s)$ being the set of traffic demand times for which a time-respecting path from *i* to *d* that finishes before $t_s$ exists. In order to reduce the computation time, $m_{i,d}^{T2T}(t_s)$ is approximated by uniformly sampling $t_k$ within $\mathcal {T}_{i,d}^*(t_s)$.

*Tendency towards Target (TTT)* The metric $m^{TTT}_{i,d}(t_s)$ considers the previous contacts between node *i* and *d* till time $t_s$. It’s inspired by the finding that if a node pair has more contacts in the past and more contacts recently, the node pair is more likely to have a contact at the next time step (Zou et al. 2023). This metric can be computed iteratively as:

5 $$\begin{aligned} m^{TTT}_{i,d}(t) = a_{i,d,t} + \alpha m^{TTT}_{i,d}(t - 1), \end{aligned}$$

with $m^{TTT}_{i,d}(0) = 0$. It increases when there is a contact between nodes *i* and *d*, and decreases over time according to its forgetting factor $\alpha$, where $0\le \alpha \le 1$. When $\alpha =1$, $m^{TTT}_{i,d}(t_s)$ counts the total number of contacts between *i* and *d* during $[1,t_s]$. When $\alpha =0$, $m^{TTT}_{i,d}(t_s)=a_{i,d,t_s}$. The distance $m^{TTT}_{i,d}(t_s)$ tends to be higher if *i* and *d* have more contacts at times closer to $t_s$.

These three distance metrics, or navigation strategies, define the distance between any node and a target node from three perspectives: how well the two nodes are connected via different paths in the aggregated network observed (RES), how well the two nodes are connected via the fastest paths in the temporal network observed in the past (*T*2*T*), and how well the two nodes interact directly with each other in the temporal network observed (*TTT*). For all three metrics, the distance $m^{X}_{d,d}(t_s) = 0$ of the destination itself is the minimal among all nodes. Whenever a hosting node has contact with the destination, the information will be forwarded to the destination.

Given a traffic demand $(s,d,t_s)$ and a navigation strategy *X*, the navigation process is as follows. First, the distance metric is computed between any node and *d* based on the temporal network observed until $t_s$. Initially, the information is hosted at *s*. At every contact involving the node hosting the information, the information is forwarded to the contacted node only if it has a smaller distance to destination *d* than the hosting node, thus becoming the only node that hosts the information. The process ends when the destination *d* is reached or all contacts till the end time *T* of the temporal network *G* have already been taken into account.

### Data

In this paper, we confine ourselves to four empirical physical contact networks (human interaction) to evaluate navigation methods: *Hospital* http://www.sociopatterns.org/datasets/hospital-ward-dynamic-contact-network/ (Vanhems et al. 2013), conference *Hypertext 2009* http://www.sociopatterns.org/datasets/hypertext-2009-dynamic-contact-network/ (Isella et al. 2011), conference *SFHH* http://www.sociopatterns.org/datasets/sfhh-conferencedata-set/ (Génois and Barrat 2018), and *High School 2011* http://www.sociopatterns.org/datasets/high-school-dynamic-contact-networks/ (Mastrandrea et al. 2015)]. In each of the networks, two individuals have a contact (their link being activated) when they interact, i.e., they are close in space. All interaction networks are constructed based on *wearable* devices that monitor face-to-face interactions. A summary of the properties of the networks can be found in Table 1. Each network has no contact for approximately 40–69% of the time, partially due to resting periods such as evenings.

**Table 1:** Basic properties of the empirical networks used

Network	*N*	*M*	*L*	*T*	${\delta }$ (s)	*p*	Type
*Hospital*	75	1139	32424	17375	20	$6.7\times 10^{-4}$	Human interaction
*Hypertext 2009 (HT09)*	113	2196	20818	10607	20	$3.1 \times 10^{-4}$	Human interaction
*SFHH*	403	9565	70261	5715	20	$1.5\times 10^{-4}$	Human interaction
*High School 2011*	126	1710	28561	13617	20	$2.7\times 10^{-4}$	Human interaction

*N* is the number of nodes, *M* is the number of pairs of nodes with one or more contacts, *L* is the total number of contacts recorded, *T* is the time span of the observation window in number of time steps, $\delta$ denotes the time resolution, which is the duration of each time step, and *p* is the probability that a random node pair has a contact at any time step

## Results

In this section, we first evaluate the performance of the different strategies proposed in Sect. 2 in the data sets previously described. Then, we study the correlation between any two distance metrics/strategies. This aims to understand whether any two metrics rank nodes similarly or not, with respect to nodes’ distance to any target at any time associated with any traffic demand. This could help us explain the (dis)similar performance between strategies. Finally, we study the stability of the ranking of nodes’ distance to a target as the time of a traffic demand varies, which may explain why some strategies perform well when the temporal network is only observed for a short period $[1,t_s]$.

### Navigation performance

**Fig. 1:**
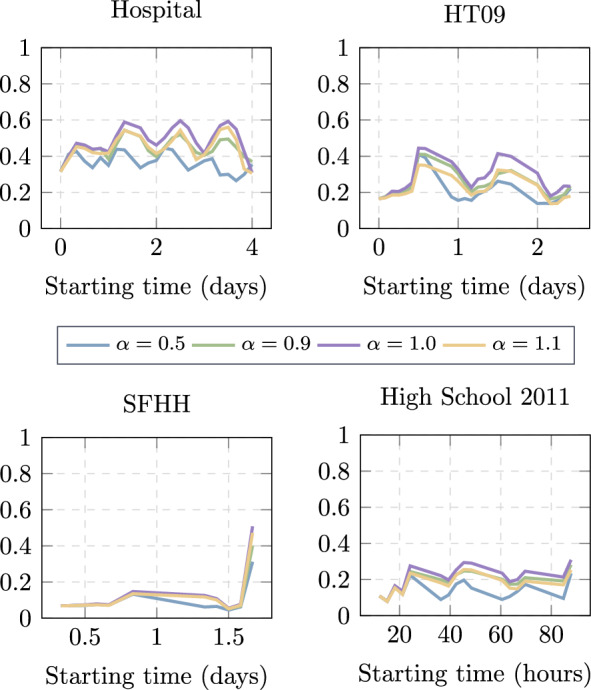
Efficiency $E_{\mathcal {G}}^{TTT}(t_s)$ of strategy Tendency towards Target (TTT) as a function of the starting time $t_s$ of traffic demands for different values of $\alpha$ in four empirical temporal networks

Since the strategy Tendency towards Target (*TTT*) has one control parameter $\alpha$, we evaluate the efficiency of strategy *TTT* with different values of $\alpha$ to identify the optimal $\alpha$ first. As shown in Fig. 1, $\alpha = 1.0$ tends to result in the highest efficiency for all possible starting times $t_s$ in all data sets. Therefore, we will compare the rest of the metrics with *TTT* using $\alpha =1$, and we will refer to the *TTT* metric with $\alpha =1$ as the *TTT* strategy for simplicity.

**Fig. 2:**
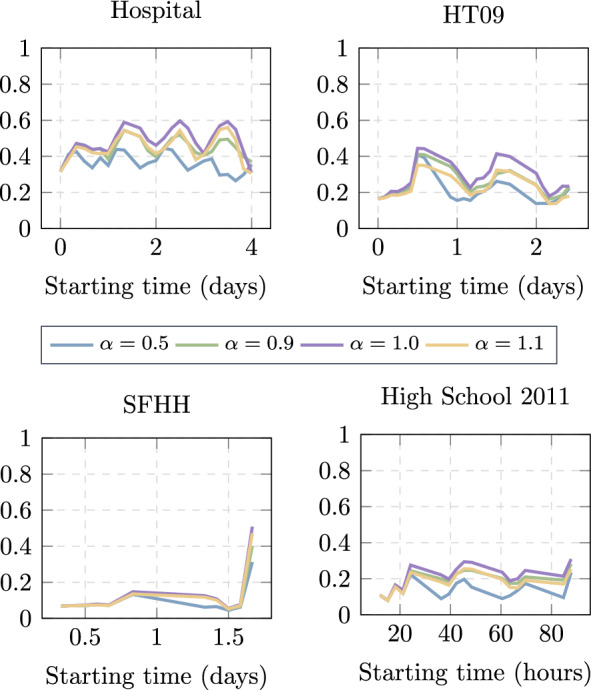
Effectiveness $\varepsilon _{\mathcal {G}}^{X}(t_s)$ of each strategy X as a function of the starting time $t_s$ of traffic demands in four empirical temporal networks

**Fig. 3:**
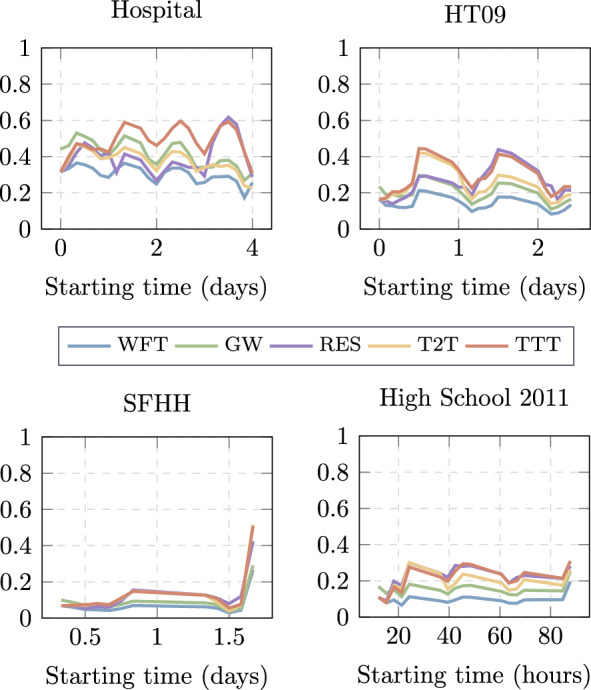
Efficiency $E_{\mathcal {G}}^{X}(t_s)$ of each strategy X as a function of the starting time $t_s$ of traffic demands in four empirical temporal networks

We measure both the effectiveness and efficiency of the three strategies proposed, along with the two baselines, in four empirical datasets. Figures 2 and 3 show the comparison between them. In every data set, all the proposed strategies improve over the Wait for Target *WFT* baseline. The other baseline, Greedy Walk (*GW*), performs the best when $t_s$ is small, i.e., when there is hardly any network information observed to estimate the distance of each node to the target node. From the three proposed strategies, *TTT* tends to perform the best more consistently along different starting times and data sets. This suggests that, in general, it is wise to forward the information to a node that has more contacts with the target node in the observation period $[1,t_s]$ than the hosting node. Furthermore, *RES* strategy tends to slightly outperform *TTT* when $t_s$ is large, at least larger than *T*/2. Strategy *RES* forwards the information to a contacted node in case the contacted node has more and shorter paths with the target than the hosting node in the observed aggregated network. A long observation period, thus a large $t_s$ tends to enhance the performance of RES. Among the four temporal networks, we observe network *SFHH* has the lowest contact density (probability *p* that a node pair has a contact at any time, shown in Table 1). Its low contact density leads to low navigation performance, so we could not distinguish the performance among the strategies considered. If a network has extremely high contact density, every node tends to have a high chance of contacting the target node at any time. This leads to the high performance of all strategies, almost the same as the optimal strategy that assumes the future network is known. Differences among the strategies in navigation can be observed in temporal networks between these two extremes.

### Correlation between metrics

**Fig. 4:**
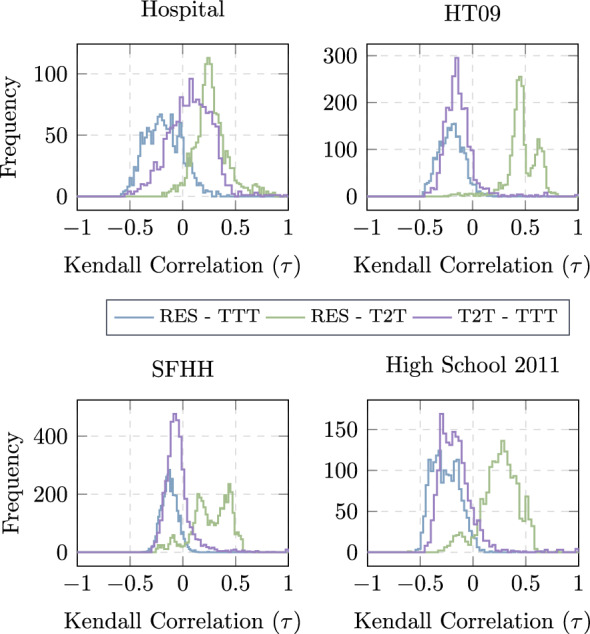
Distribution of the correlation of metrics for different strategies

We study further how the proposed distance metrics are related to each other. The objective is to verify whether these metrics indeed capture different network properties as intended when defining them, which could explain their different performance in navigation. Given a traffic demand $(s,d,t_s)$, the distance of each node to the target *d* can be computed according to each distance definition *X* based on the network observed till $t_s$ and recorded as a distance vector ${\textbf{m}}_d^{X}(t_s)$. We compute the *Kendall rank correlation* Kendall (1938) between two distance metrics, namely the two corresponding distance vectors for each combination of destination *d* and starting times $t_s$. The distribution of the correlation considering different destinations *d* and starting times $t_s$ is shown in Fig. 4 for each data set. The choice of Kendall rank correlation is motivated by the following. Given a traffic demand $(s,d,t_s)$, the routing decision, to forward the information or not when a hosting node has a contact, depends only on whether the contacted node has a smaller distance to the destination or not than the hosting node. Hence, the routing decision based on any distance metric, depends only on the ranking of nodes in the distance vector. Kendall rank correlation between two distance metrics naturally indicates the tendency that these two metrics/strategies lead to the same routing decision. The correlation between any two metrics, as shown in Fig. 4, is in general low, suggesting that each metric captures a different property, or distance perspective between two nodes. This observation also indicates that the proposed strategies are supposed to perform differently, as long as the temporal network does not belong to the two extreme cases discussed above.

### Rank stability

**Fig. 5:**
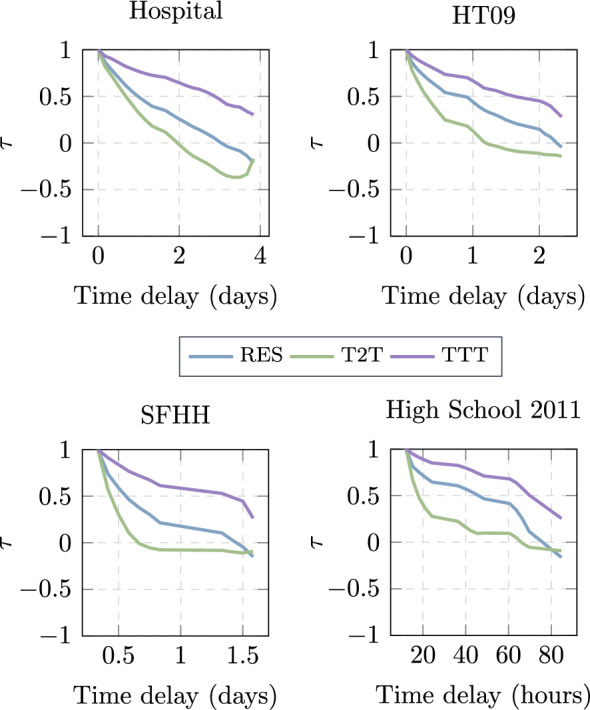
Average Kendall correlation $\tau$ between a distance metric at two times with given a time delay

We explore further whether the ranking of nodes with respect to their distance to a given target *d* derived from each distance metric is stable as $t_s$ increases. In other words, we wonder whether the ranking of nodes in the distance vector $\textbf{m}_d^{X}(t_s)$ changes with $t_s$. In order to answer this question, we explore the average Kendall rank correlation between $\textbf{m}_d^{X}(t_s)$ and ${\textbf{m}}_d^{X}(t_s-\Delta t))$ over all possible targets *d* and times $t_s$, i.e.,

6 $$\begin{aligned} \tau ^{X}(\Delta t) = \dfrac{1}{N \cdot (T-\Delta t)} \sum _{d} \sum _{t_s=\Delta t}^{T} \tau ({\textbf{m}}_d^{X}(t_s), \textbf{m}_d^{X}(t_s-\Delta t)), \end{aligned}$$

where *N* is the number of nodes, *T* is the length of the time window, *X* is the relevant metric, $\Delta t$ is the time delay, and $\tau (x,y)$ represents Kendall’s $\tau$ between vectors *x* and *y*. The results are represented in Fig. 5 for all delays and metrics per each data set.

In general, the correlation decays as the time delay increases. We observe that the Tendency towards Target (*TTT*) strategy is the most stable or decays the least over time. For such a stable metric, the distance vector ${\textbf{m}}_d^{X}(t_s)$ is not needed to be updated frequently to route demands towards *d* at different times. The stability of tendency-towards-target (*TTT*) also supports the reasonably good performance of *TTT* when the starting time is small, i.e., when the temporal network has been observed for a short period.

## Conclusions

In this paper, we have studied the problem of navigation in temporal networks based on the temporal network observed in the past. We have proposed three distance metrics or routing strategies based on different topological and temporal properties of the network and considered two benchmark strategies. These navigation methods are evaluated in physical contact networks from the perspectives of efficiency, i.e., how fast information can be transported, and effectiveness, i.e., the probability that the target can be reached, both compared with the optimal solution when the temporal network in the future is known. These distance metrics capture different properties between nodes, supported by their definitions as well as the relatively low correlation among them.

Among all the strategies proposed, we found that the Tendency towards Target (*TTT*) performs the best overall, even when the starting time of a traffic demand is early; thus, temporal network observed for routing has a short duration. This can be partially explained by the stability of the metric in ranking nodes over time. When the starting time is late, the Effective Resistance (*RES*), which takes into account all paths between each node pair in the aggregated network of the temporal network observed, tends to slightly outperform *TTT*. It is worth noting that the best performance of *TTT* was achieved by using a parameter $\alpha = 1.0$, which means the distance metric from a node to the target is simply the number of contacts between the two nodes that have been observed in the past. In practice, only the number of contacts between each node pair needs to be recorded over time until a new traffic demand is launched. When the hosting node has a contact with a node that has more contacts with the target, the information is forwarded. Hence, the *TTT* strategy is, in general, efficient, effective, and of low cost.

The design of the two best-performing strategies *TTT* and *RES* utilizes the heterogeneous distribution of inter-event times between consecutive interactions (burstiness) and of the number of interactions between a node pair. The distance metric of *TTT* estimates the tendency of future connections between two nodes by considering both the frequency and timing of their previous interactions. The distance metric of *RES* takes into account not only the number of contacts between a node pair, but also how well the node pair is connected by paths. These two examples could inspire the development of novel navigation strategies that integrate burstiness and various forms of heterogeneity in temporal networks. Moreover, burstiness and heterogeneity have been mostly observed in physical contact networks, not necessarily in other types of networks. In this work, we have only considered physical contact networks, and the results are therefore limited to them. Studying how navigation methods perform and could be redesigned in other types of temporal networks is an interesting future research question. This will also unravel how the properties of a temporal network affect the performance of routing strategies.

## Data Availability

The datasets used are publicly available. More information can be found in the corresponding references.
